# Towards controlled and simple design of non-enzymatic amperometric sensor for glycerol determination in yeast fermentation medium

**DOI:** 10.1007/s00216-024-05316-7

**Published:** 2024-05-03

**Authors:** E. V. Zolotukhina, E. V. Butyrskaya, C. Fink-Straube, M. Koch, Y. E. Silina

**Affiliations:** 1 Federal Research Center of Problems of Chemical Physics and Medicinal Chemistry, Russian Academy of Sciences, Chernogolovka, Moscow Region Russia; 2 Department of Chemistry, Voronezh State University, Voronezh, Russia; 3 INM – Leibniz Institute for New Materials, Saarbrücken, Germany; 4 Institute of Biochemistry, Saarland University, Campus B 2.2, Room 317, Saarbrücken, Germany

**Keywords:** Palladium deposits, Glycerol electrooxidation, Quantification, Adsorption, Interfering species, Yeast fermentation medium

## Abstract

Glycerol is a widely used signaling bioanalyte in biotechnology. Glycerol can serve as a substrate or product of many metabolic processes in cells. Therefore, quantification of glycerol in fermentation samples with inexpensive, reliable, and rapid sensing systems is of great importance. In this work, an amperometric assay based on one-step designed electroplated functional Pd layers with controlled design was proposed for a rapid and selective measurement of glycerol in yeast fermentation medium. A novel assay utilizing electroplated Pd-sensing layers allows the quantification of glycerol in yeast fermentation medium in the presence of interfering species with RSD below 3% and recoveries ranged from 99 to 103%. The assay requires minimal sample preparation, viz. adjusting of sample pH to 12. The time taken to complete the electrochemical analysis was 3 min. Remarkably, during investigations, it was revealed that sensitivity and selectivity of glycerol determination on Pd sensors were significantly affected by its adsorption and did not depend on the surface structure of sensing layers. This study is expected to contribute to both fundamental and practical research fields related to a preliminary choice of functional sensing layers for specific biotechnology and life science applications in the future.

**Graphical Abstract:**

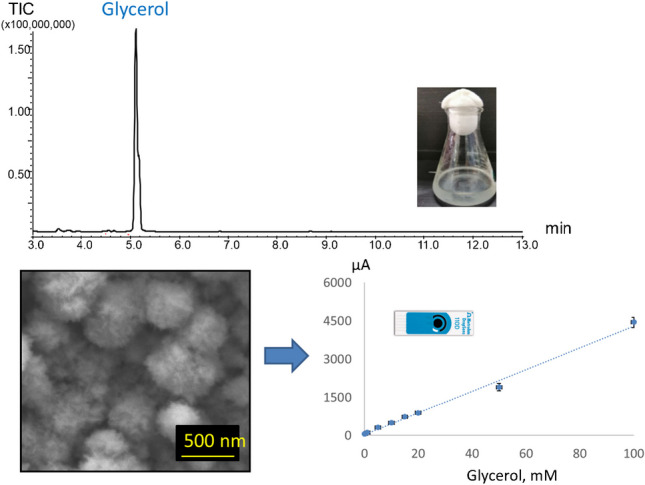

**Supplementary Information:**

The online version contains supplementary material available at 10.1007/s00216-024-05316-7.

## Introduction

Glycerol is one of the major metabolites of ethanol fermentation by *Saccharomyces cerevisiae* [1, 2]. Glycerol is also used as an important signaling biomarker in medicine, e.g., for clinic diagnostics and control of triacylglycerides level [3].

The standard analytical methods of glycerol analysis, including liquid and gas chromatography [4, 5], are disadvantageous since they require highly qualified personnel, expensive equipment, and complex multi-step sample pretreatment, i.e., derivatization [6]. However, the application of derivatization for real objects, viz. fermentation media, can lead to co-derivatization of other compounds present in samples that impacts the bioanalytical significance of the obtained results.

Traditional enzymatic approaches for glycerol determination in case of dehydrogenase [7, 8] or oxidase biochemical reactions showed low selectivity, sensitivity, and moderate shelf and life time [9, 10]. Moreover, high cost, low activity of the intact enzymes, and low linear dynamic range (e.g., 1–2 mM) [8] significantly limit the laboratory application of enzymatically based approaches [11]. At the same time, the concentration of glycerol in real fermentation samples can readily exceed the value above 100 mM [12] at which enzymatic biosensors cannot be active. Therefore, the development of robust, selective, simple, rapid, and cheap assays for glycerol determination and control in biological and environmental samples remains an actual challenge [13].

As a desirable alternative tool for a rapid glycerol detection, non-enzymatic electrochemical sensing could be considered [13–17]. However, the electrooxidation of glycerol on the surface of a conventional glassy carbon or noble metal-based electrodes [18] practically occurs at over-potentials (viz. higher than 1 V) that significantly reduces the selectivity of its determination, particularly in real samples [15].

Another problem associated with amperometric non-enzymatic glycerol sensing is related to the complex design of functional layers of electrodes. Functional sensing layers can consist of more than 3–4 components: for example, a glassy carbon electrode with electrochemically reduced graphene and electrochemically deposited gold nanoparticles (GCE-ErGO-EAuNp) [19], copper oxide nanoparticles supported on multi-walled carbon nanotubes/pectin composite (CuONP/Pe(MWCNT)/GC) [14], graphene oxide nanoparticle decorated pencil graphite electrode (GKNPs/GPONPs/GrONPs/PGE) [17], and ammonia-etched platinum-palladium-silver (PtPd@Ag-NH_3_) films [18] were reported.

From an engineering point of view, a complex design of functional layers of electrodes makes their synthesis and the subsequent electroanalytical performance highly irreproducible. Therefore, to support a reliable synthesis route of electrodes as well as their reproducible analytical characteristics during glycerol sensing, a simple design of their functional layers should be maintained [20]. In this aspect, an approach allowing producing sensors containing a single functional component with a reproducible design and tuned structure would be a desirable alternative.

Regarding the electrooxidation mechanism of alcohols, it relies on the cleavage and transport of the hydrogen atom from the alcohol molecule adsorbed on the electrocatalyst [21–24]. Palladium (Pd) can serve as an efficient catalyst to facilitate hydrogen absorption [25, 26]. Therefore, palladium is of particular interest in the field of amperometric sensor development for non-enzymatic sensing of alcohols, viz. glycerol.

In the present work, several designs of one-step produced electroplated Pd particle–modified electrodes for glycerol determination in yeast fermentation medium were developed. The electroanalytical performance of the proposed sensors with regard to a linear dynamic range, sensitivity, stability, and interference behavior was evaluated and the obtained analytical merit was highlighted. The performance of the proposed sensors with a simple and highly reproducible surface in real fermentation samples was validated by using commercial Pd-ink-modified electrode and gas chromatography-mass spectrometry (GC–MS). More significantly, for the first time, it was shown how the adsorption of glycerol on Pd layers can be specific in the presence of interfering species of yeast fermentation medium.

This study is expected to contribute to a rational design of Pd electrocatalytic layers based on the tuning of surface properties to eventually enhance the electrochemical signal towards efficient glycerol detection and quantification in multiple mixtures.

## Experimental part

### Chemicals and materials

DRP-110DGPHOX screen-printed electrodes (SPEs) were obtained from DropSens (Metrohm, Germany). The electrodes were printed on ceramic substrates. Each sensor consisted of a carbon working electrode (WE) (modified with graphene oxide), a carbon counter electrode (CE), and a silver reference electrode (RE). The diameter of the working electrode was 0.4 cm. PdCl_2_, glycerol solution (85%), ethanol, EtOH, butanol, BuOH, KOH pellets, derivatization agent N-trimethylsilyl-N-methyl trifluoroacetamide (MSTFA), and yeast fermentation HC medium (Hartwell’s Complete medium) supplemented with 10% of yeast nitrogen base (YNB) were received from Merck (Darmstadt, Germany). Organic-free, deionized (DI) water was generated by an Elga PureLab (Celle, Germany) water purification system.

### Yeast fermentation

Yeast cells (strain By4742) were streaked from the stock onto agar plates and cultivated at 30 °C in the incubator for 24 h. The grown colonies were transferred to another flask filled with 10 mL of a fresh HC medium to complete the growth process at aerobic conditions. The cultivation time was ranged from 24 to 72 h. Depending on the cultivation time, the obtained cells had an optical density (OD) between 3.0 ± 0.4 and 6.5 ± 0.3. OD was measured at 600 nm on a cell density meter (Ultrospec 10, Fisher Scientific).

After cultivation and measurement of OD, cells were removed by centrifugation carried out at 13,000 rpm for 10 min at 20 ± 2 °C. The obtained supernatants were used for glycerol sensing. For quantitative analysis, the standard addition approach was used in all experiments. Prior to electrochemical analysis, pH of the samples was set to 12 by KOH pellets and controlled by a portative pH meter (Horiba LAQUAtwin).

### Formation of Pd-functional layer on electrodes

Commercial Pd-ink-modified electrodes were used in their original form without further modification. Pd particles were formed in a droplet mode on the SPE surface during galvanostatic deposition at a cathodic current of − 2.5 mA for 30 s (Sensor 1) from the acidic PdCI_2_-contaning electrolyte (10 mg/mL, pH 2). Briefly, 10 µL of the electrolyte solution was placed on the surface of the working electrode (WE) followed by a subsequent cathodic polarization (anode – Pd wire) [27]. After palladium electrodeposition, the electrode surface was carefully washed using DI water. The prepared sensor was stored at ambient conditions prior to electrochemical studies.

To compare the analytical merit of the produced Sensor 1, several analogues at higher cathodic currents, i.e., − 6 mA and longer deposition times (120 s and 240 s), were produced from the same electrolyte in a droplet mode (Sensor 2 and Sensor 3, respectively).

The reproducibility of the synthesis route for the synthesized functional layers was evaluated as a batch-to-batch reproducibility of the baseline of Pd-modified sensors in model solutions at pH 12.

Calculation of the electroactive surface area (ECSA) of electrodes was performed via evaluation of the oxygen desorption peak charge values in cyclic voltammetry (CV) mode according to a previous study [28]. For the estimation of specific ECSA (m^2^/g), the Pd weight (µg) was evaluated by Faraday’s law from the charge passed during dissolution of Pd deposits from the WE in 1 M HCl in a chronopotentiometry mode according to a protocol reported in [29]. Different anodic currents were applied depending on the size of the Pd particles: the dissolution of small particles (Sensor 1) was conducted at 0.1 mA; bigger particles (Sensor 2 and Sensor 3) were dissolved at 0.3 mA. The dissolution of Pd-ink from the commercial electrode was conducted at 0.5 mA.

### Electrochemical studies

The electrochemical performance of Pd particle–modified sensors in modeled aqueous solutions and yeast fermentation medium/supernatants was explored at pH 12 in a droplet mode (150 µL of the droplet was placed over the surface of all three electrodes, e.g., WE, CE, and RE) via CV in the range of − 0.4 V …0.8 V at 20 mV/s on a one-channel biologic Potentiostat PalmSens4 (PalmSens, Utrecht, The Netherlands). All measurements from the same electrode were recorded at least in triplicate. For data acquisition, the second scans were utilized. The response during calibration of electrodes with Pd particles was recorded in amperometric AM mode at the applied voltage equal to 0.60 V (unless otherwise not specified).

### Scanning electron microscopy (SEM/EDX)

The morphology of SPEs was studied by scanning electron microscopy (SEM) on a FEI (Hillsboro, OR, USA) Quanta 400 FEG system equipped with an EDAX (Mahwah, NJ, USA) Genesis V 6.04 X-ray module. Secondary electron images were acquired in high vacuum mode using the Everhart–Thornley detector at an accelerating voltage of 10 keV.

### Gas chromatography-mass spectrometry (GC–MS)

Chromatographic analysis of fermentation samples was performed on a QP5050 GC–MS system (Shimadzu, Japan) equipped with a PAL auto sampler (CTC Analytics, Zwingen, Switzerland). Prior to GC–MS analysis, samples were treated by MSTFA according to a previously reported protocol [5]. Briefly, the aqueous phase was evaporated from the 200 μL of the received samples/supernatants under N_2_ atmosphere followed by the subsequent addition of 40 μL of MSTFA. Next, the suspension was heated at 60° C for 30 min. The received solutions were used for the subsequent GC–MS analysis. Injection volume of the sample into the GC-inlet was 0.5 µL.

GC–MS separation was carried out in a split mode 1/15 on a ZB-5HT Inferno column (30 m × 0.25 mm, thickness 0.25 µm) at a purge flow of 1 mL/min at the following gradient conditions: starting temperature was 50 °C held for 2 min, then increased to 300 °C at 25 K/min and held at 300 °C for 1 min. The total analysis time was 13 min. The injection temperature was set at 200 °C. Mass spectra were recorded in TIC mode in the range of *m/z* 100–500. MS interface was set at 250 °C and ion source temperature at 200 °C.

### Quantum-chemical calculations

To study the impact of the adsorption stage on glycerol electrooxidation at Pd-modified electrodes, quantum-chemical studies were carried out by density functional theory (DFT) B3LYP using Gaussian 09 program. The methods of DFT, among modern methods of quantum chemistry, propose excellent modeling accuracy at the optimal computing resources spent.

The 6-31G(d,p) basis set was used for O, N, and H atoms [30]. LANL2DZ ECP basis with the added f-polarization function was used for Pd. The applied DFT allows to predict possible molecular interactions in the target systems [31], viz. the adsorption parameters of glycerol on the framework of metal clusters [32].

During the calculations, the position of glycerol was varied on Pd and PdO fragments (formed during electrooxidation, anodic range of potentials). The total Gibbs adsorption energies (G_ads_) of glycerol and EtOH at Pd and PdO clusters were calculated based on an earlier reported approach [29].

## Results and discussion

In our previous work, commercial Pd-ink-modified electrodes with a diameter of functional electroactive particles of 600–800 nm were used to screen glycerol in a cultivation medium of bacterial cells (*Escherichia coli*) [6]. However, the electroanalytical performance of Pd-ink-based electrodes was reduced due to a non-electroactive polymer layer utilized in their design. The use of a polymer as a binding agent was justified by the applied drop coating methodology to form functional sensing layers.

Here, as a possible alternative to commercial Pd-ink-modified electrodes, we used electroplating of Pd particles [27] from PdCl_2_-contaning electrolyte allowing to obtain Pd deposits in their refined form, e.g., without polymers or other non-electroactive substances (i.e*.*, phosphates, ammonium complexes, etc.), ESI, Fig. S1 (shown for Sensor 3 as a case study).

### Characterization of electroplated Pd particle–modified sensors

**Fig. 1:**
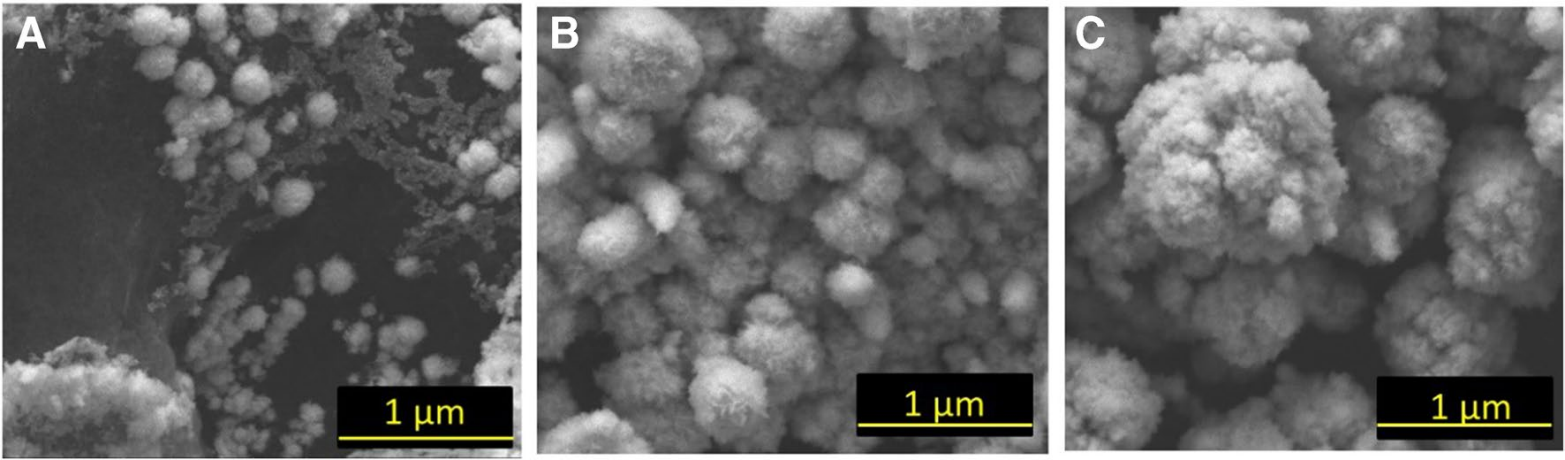
SEM images of sensors modified by electroplated Pd deposits synthesized from the acidic PdCl_2_-based electrolyte at various experimental conditions: **A** at − 2.5 mA for 30 s; **B** at − 6 mA for 120 s; **C** at − 6 mA for 240 s

First, the impact of synthesis parameters, e.g., the applied cathodic current and deposition time on the architecture of electroplated Pd-functional layers of sensors, was investigated. SEM studies highlight the formation of Pd deposits with a size between 100–200 nm (Sensor 1), 600–800 nm (Sensor 2), and 1.0–1.5 µm (Sensor 3) (Fig. 1).

The morphology of electroplated Pd particles indicates that larger globules were formed by aggregation and overgrowth of the individual smaller structures with increase of a current and deposition time. It means that the synthesis route of Pd particles can readily be instrumentally controlled. This is especially important to support a reproducible architecture of functional sensing layers. Only a reproducible design of sensing layers can guarantee reproducible and reliable electroanalytical performance of amperometric sensors during subsequent electrochemical analysis of a target analyte. Notably, relative standard deviation (RSD) for all electrodes with electrodeposited Pd particles did not exceed 10% (Table 1). In contrast, the reproducibility of the baseline of Pd-ink-modified electrode reached the level of 31% that is most likely explained by the used preparation approach of functional layers (drop coating methodology).

**Table 1:** Electrochemical characterization and reproducibility of the baseline (row data were extracted from the second scans of three batches of each sensor) of sensors modified by Pd particles (polarized from − 0.4 to 0.8 V at 20 mV/s in buffer, pH 12)

Electrode	Reproducibility of the baseline		Mass, µg	ECSA**, cm^2^	ECSA, m^2^ g^−1^
	Baseline, µA ± SD	RSD, %			
Sensor 1^*^	124 ± 3	2.0	25 ± 3	2.7	10.8
Sensor 2	136 ± 5	3.5	95 ± 5	2.1	2.2
Sensor 3	242 ± 34	9.3	115 ± 3	2.9	2.5
Commercial Pd ink	356 ± 111	31.1	124 ± 34	9.2	7.4

^*^Data acquisition was conducted at 0.55 V

^**^For ECSA calculation, sensors were polarized in buffer at pH 7 from − 0.4 to 0.4 V

The obtained SEM data were in line with the calculated mass of electroplated Pd deposits (Table 1). As expected, the increase of the deposition current and time leads to the increase of deposited palladium weight from 25 ± 3 µg (Sensor 1) to 115 ± 3 µg (Sensor 3). At the same time, ECSA decreased in a row: from 10.8 m^2^g^−1^ defined for Sensor 1 to 2.5 m^2^g^−1^ found for Sensor 3. Hence, it can be expected that the analytical response for Pd-modified electrodes with the highest ECSA should have an advanced performance. At the same time, glycerol oxidation process demonstrates a complicated mechanism with a participation of the adsorbed oxygenic species and intermediates [33, 34]. Therefore, the analytical merit of the small Pd particles is not so obvious and cannot be predicted [35].

### Figures of merit of one-step electroplated Pd particle–modified sensors

To make the recorded trends more pronounced, the electroanalytical performance of the produced sensors modified by electroplated Pd particles was examined in model solutions at high concentrations of glycerol. The obtained analytical merit was compared with commercial Pd-ink-based electrodes.

**Fig. 2:**
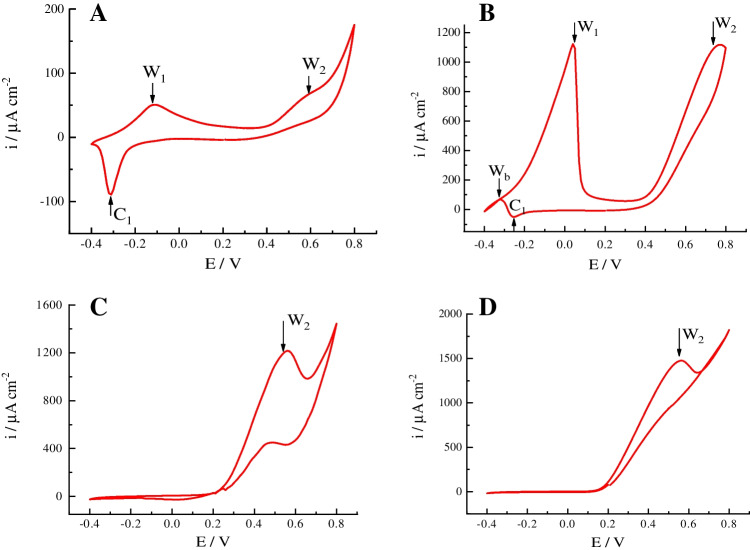
CV plots recorded from Pd-based sensors at 20 mV/s in 100 mM of model glycerol solution at pH 12: **A** commercial Pd-ink-modified electrode, **B** Sensor 1, **C** Sensor 2, **D** Sensor 3

The electrochemical behavior of Pd sensors differs depending on the used preparation mode (Fig. 2). Briefly, commercial Pd-ink-modified electrodes and electroplated Pd-based electrode (Sensor 1) demonstrated two anodic peaks, W_1_ and W_2_ (at approx. − 0.1 V and 0.6 V), corresponding to the glycerol oxidation process (i.e., these peaks are absent in background solution) on reduced (− 0.1 V [6]) and oxidized (0.6 V) Pd surfaces, while Sensors 2 and 3 with larger Pd particles showed only one anodic peak, W_2_, at 0.6 V. The cathodic peak that corresponded to the oxygen reduction on the Pd surface was visible for Pd-ink-modified electrode and Sensor 1 and absent for Sensor 2 and Sensor 3. In the case of Sensor 1, the cathodic process of reduction of the adsorbed oxygen interfered with the anodic process during backward scan (wave W_b_) that leads to a compromise current in this potential range. The latter effect of anodic and cathodic current superposition is typical for alcohol oxidation processes on Pd [6].

In terms of the analytical merit, the onset potential for the second anodic wave is increased in the row: Sensor 3 < Sensor 2 < Sensor 1 < Pd-ink sensor, corresponding to 0.18 V, 0.22 V, 0.39 V, and 0.43 V, respectively (Fig. 2, see also ESI, Fig. S2)*.* It means that the oxidation of glycerol species at 0.6 V is facile on the large Pd particles formed via electrodeposition synthesis approach. Thus, as it was supposed above, the complicated mechanism of glycerol oxidation leads to leveling the role of the specific ECSA of sensing Pd layers (Table 1).

Moreover, as it is typical for the adsorption processes, the electrochemical behavior of sensors with small and large Pd particles depends on glycerol concentration. Thus, with the increase of glycerol concentration from 1 to 100 mM, the anodic peak W_2_ potential of Sensor 1 shifted to higher values and the anodic peak currents at − 0.1 V (W_1_) and 0.6 V (W_2_) are growing while the current of the cathodic peak, C_1_, decreased (ESI, Fig. S3). It means that the overpotential of W_2_ for glycerol oxidation increases for Sensor 1 from 0.50 to 0.79 V with the increase of glycerol concentration from 1 to 100 mM, respectively. These data were in accordance with a mechanism of alcohol oxidation on palladium in alkaline media, viz. participation of oxygenic species (OH^−^, O_ads_, OH_ads_, etc.) [34]. When the concentration of OH-ions in solution is comparable or lower than the amount of glycerol, the deficit of oxygenic reaction species leads to an overpotential of the reaction.

**Fig. 3:**
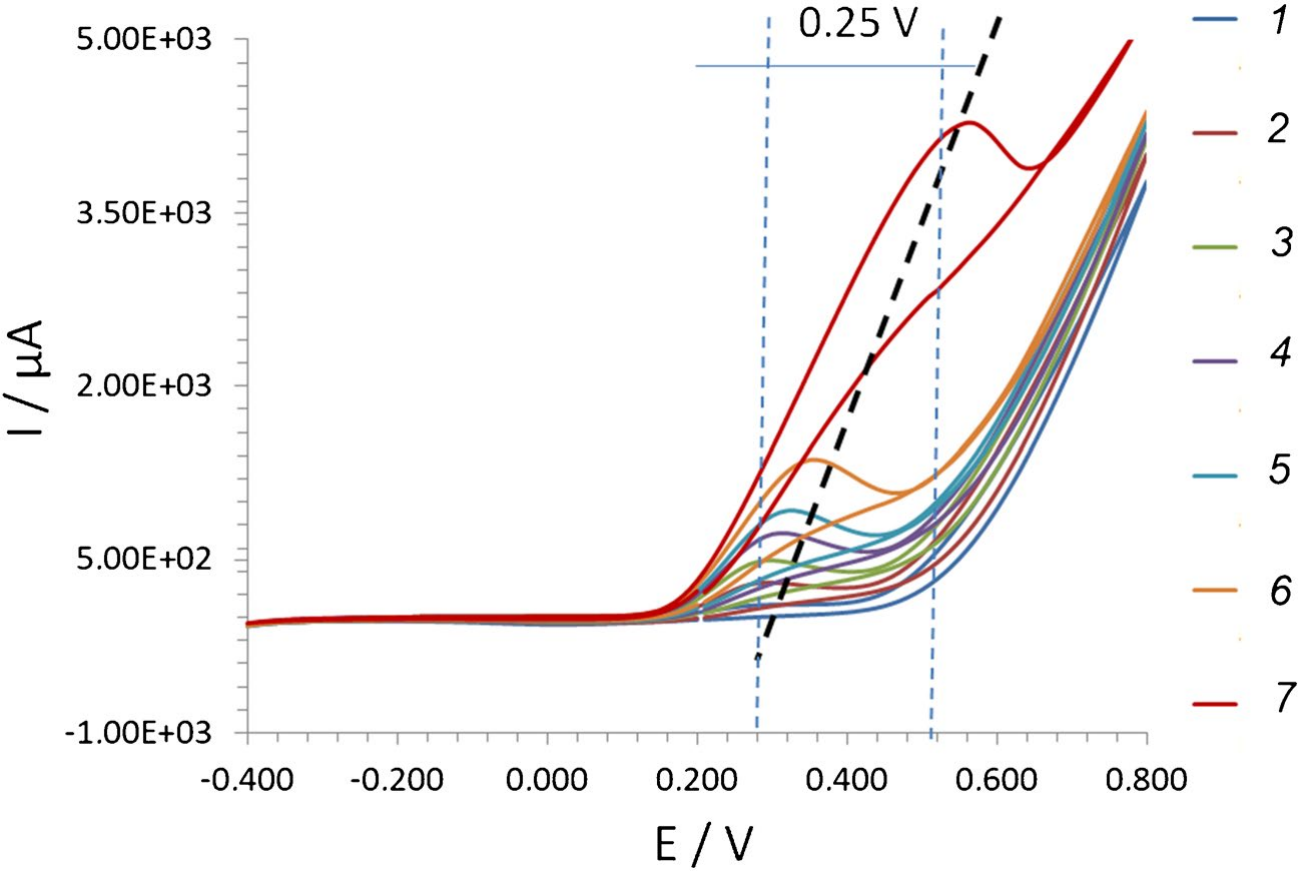
CV plots recorded at 20 mV/s in model glycerol solution at pH 12 from Sensor 3: 1, 2, 3, 4, 5, 6, 7 — 1 mM, 5 mM, 10 mM, 15 mM, 20 mM, 50 mM, 100 mM, respectively

In contrast, both Sensor 2 and Sensor 3 with the large Pd particles demonstrate unusual properties. For Sensor 2, the first anodic peak W_1_ at low potential and cathodic peak C_1_ disappeared with the increase of glycerol concentration; at the same time, the anodic wave W_2_ at 0.6 V practically did not depend on glycerol concentration (ESI, Fig. S3). For Sensor 3, the first anodic W_1_ and cathodic peaks C_1_ are absent while the second anodic peak W_2_ potential increased from 0.3 to 0.6 V with the increase of glycerol concentration from 1 to 100 mM (Fig. 3).

The obtained results confirmed that the electrooxidation of glycerol is induced by oxygenic species (O_ads_, OH_ads_, etc.) on the surface of the functional electroactive layer [34]. Apparently, with the increase of glycerol concentration, there are not enough oxygen groups on the surface of the electrocatalyst. However, the adsorption energy of oxygen and glycerol species can be different on Pd particles of various sizes. It means that the exact mechanism of glycerol electrooxidation on Pd particles is still under discussion and needs to be clarified.

Since the second anodic peak W_2_ was present in CV regardless of the applied glycerol concentration, this peak could be used as an analytical read-out range for the comparison of the analytical merit of tested sensors (see next section).

### Calibration and validation in model aqueous solutions

The read-out of the signal from Pd-ink-modified electrode, Sensor 2, and Sensor 3 in model glycerol solutions at pH 12 was conducted at 0.55 V in the whole concentration range of glycerol (Table 2). In contrast, glycerol quantification by Sensor 1 with the appropriate regression coefficient (≥ 0.98) could be conducted at 0.55 V only in the concentration range between 20 and 100 mM. To support glycerol quantification by Sensor 1 below 20 mM, the signal read-out had to be carried out at 0.3 V.

**Table 2:** Electroanalytical performance of Pd-modified sensors in model glycerol solutions at pH 12

Electrode	Calibration formula in model solutions	*R*^2^	Sensitivity*, µA·mM^−1^·cm^−2^	LDR**, mM	LOD***, mM
Sensor 1	*y* = 38.2·*x* + 154	0.9976	14.1	0.1–20	0.1
	*y* = 10.9·*x* + 696	0.9999	4.0	20–100	10
Sensor 2	*y* = 23.0·*x* + 303	0.9867	11.0	0.2–100	0.1
Sensor 3	*y* = 42.7·*x* + 41	0.9969	14.7	0.2–100	0.1
Commercial Pd-ink	*y* = 5.7·*x* + 252	0.9959	0.6	10–100	5

*Sensitivity was evaluated in LDR; ECSA values are summarized in Table 1

**LDR, linear dynamic range

***LOD, limit of detection

**Fig. 4:**
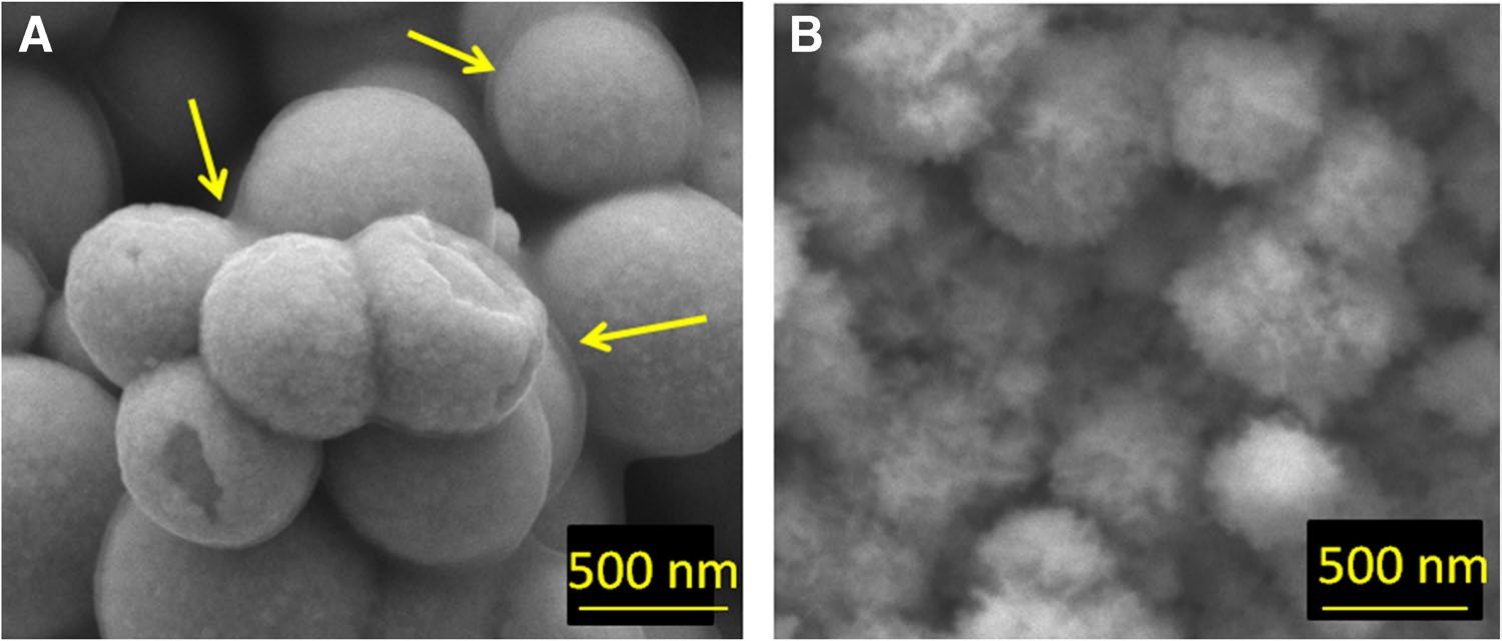
SEM images of Pd-ink functional layer (**A**) and electroplated Pd particles produced at − 6 mA for 120 s from the acidic electrolyte (**B**). *Note*: the organic polymer layer used as a binding agent is shown in (**A**) by arrows

The significant increase of a slope in the calibration formula of electroplated Pd particles versus Pd-ink (Table 2) can readily be explained by the absence of non-electroactive compounds such as polymer binding agents in the design of sensing layers (Fig. 4). Therefore, despite having almost the same dimensional factor (e.g., 500–800 nm), the electroplated Pd particles appear to be more efficient versus Pd-ink. It also explains the enhanced LDRs and sensitivity values obtained for the sensors modified by electroplated Pd particles (see Table 2).

Taken together, our data suggest that by electroplating and surface engineering, it is readily possible to achieve the controlled design of sensing layers with the advanced analytical merit, viz. LDRs and sensitivity. More significantly, the sensitivity of glycerol electrooxidation does not significantly depend on the design of electroplated Pd deposits and could be a function of glycerol adsorption features (see “Impact of the adsorption stage on the glycerol electrooxidation”).

Next, to demonstrate the reliability of the developed sensors with electroplated Pd particles for quantification of glycerol, the recovery was evaluated by standard addition approach in model solutions. For this goal, model phosphate-containing solutions with pH 12 were spiked with different glycerol concentrations (5.00, 10.00, and 100 mM). A droplet of 150 μL was then placed on the surface of sensors. After each measurement, the sensors were rinsed by DI water prior to the next run. The time taken to complete a single run was 3 min. The novel amperometric sensors with electroplated Pd deposits achieved satisfactory recovery yields ranging from 96 to 107% with an RSD of less than 5% (Table 3).

**Table 3:** Selected recovery data for glycerol determination in model solutions

Electrode/calibration formula used	Added glycerol, mM	Found glycerol, mM	Recovery, %	RSD, %
Sensor 1 *y* = 38.2·x + 154	5.00	4.80 ± 0.17	96.00	3.61
	10.00	9.83 ± 0.25	98.30	2.55
	50.00	50.50 ± 2.30*	101.00	4.49
Sensor 2 *y* = 23.0·x + 303	5.00	4.85 ± 0.07	97.00	1.55
	10.00	9.46 ± 0.22	94.60	2.41
	50.00	48.26 ± 1.24	98.78	2.53
Sensor 3 *y* = 42.7·x + 41.8	5.00	4.93 ± 0.06	98.60	1.23
	10.00	10.70 ± 0.37	107.00	3.30
	50.00	49.61 ± 1.31	99.22	2.37

*Calculated based on the formula obtained in LDR between 20 and 100 mM, *y* = 10.9·*x* + 696

### Impact of the adsorption stage on the glycerol electrooxidation

As highlighted above, the sensitivity of glycerol electrooxidation did not depend on the design of Pd deposits (see Table 2). Therefore, it was hypothesized that the sensitivity of electroplated Pd-sensing layers can be connected with the adsorption stage of glycerol. The adsorption energy will also impact the binding efficiency and surface concentration of the electroactive form of the analyte.

**Fig. 5:**
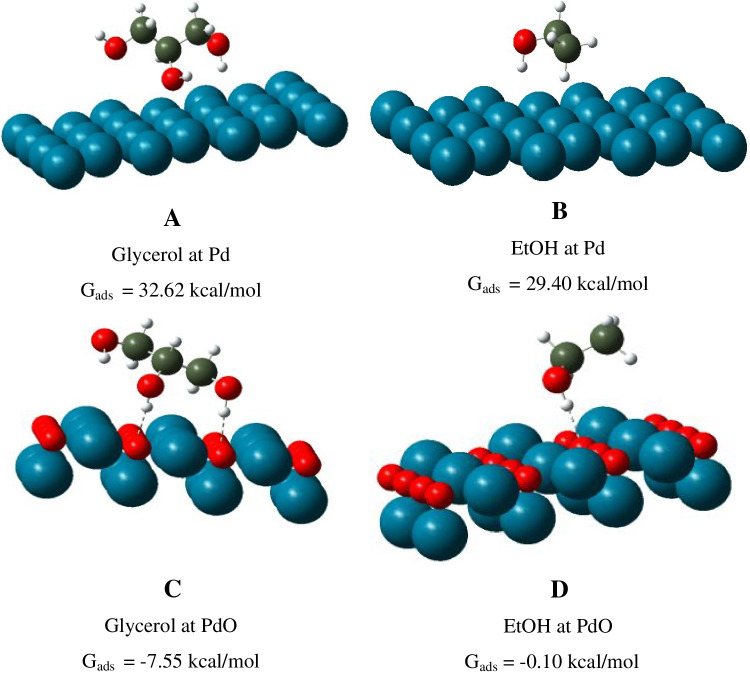
The Gibbs adsorption energies (G_ads_) and the most favorable location of glycerol (**A**, **C**) and EtOH (**B**, **D**) on Pd and PdO

Next, Gibbs adsorption energies for glycerol in comparison with EtOH were calculated at the Pd and PdO plane using DFT. As it is seen from Fig. 5, the Gibbs adsorption energy is negative for PdO plane and positive for Pd. It means that the presence of oxygenic species might influence glycerol adsorption.

It should also be mentioned that the received value of glycerol adsorption (G_ads_) on the PdO surface was almost 75 times more advantageous as compared to EtOH (Fig. 5). In addition, the adsorption of glycerol was accompanied by the formation of two hydrogen bonds. In contrast, the adsorption of EtOH proceeds with generation of a single hydrogen bond.

It means that for the efficient glycerol attachment (that is important for its subsequent electrooxidation), at least two active sites on the PdO surface are necessary. Hence, the absence of the oxygen reduction peak for Sensors 2 and 3 could be explained by the consumption of adsorbed oxygen in the glycerol electrooxidation process.

### Electroanalytical performance of electroplated Pd sensors in real yeast fermentation medium

#### Is glycerol really present in yeast fermentation medium?

Depending on the medium type, adjusted biotechnological process, line of the used yeast cells, their cultivation times and cultivation conditions, optical density of cells, etc., the amount of glycerol in fermentation medium can be very different. Thus, glycerol can be formed in cultivation medium as a product or it can be consumed by the cells as a carbon source during their physiological growth. Therefore, to verify the presence of glycerol in fermentation samples used in our study, a GC–MS protocol including derivatization of samples with MSTFA was applied [6].

**Fig. 6:**
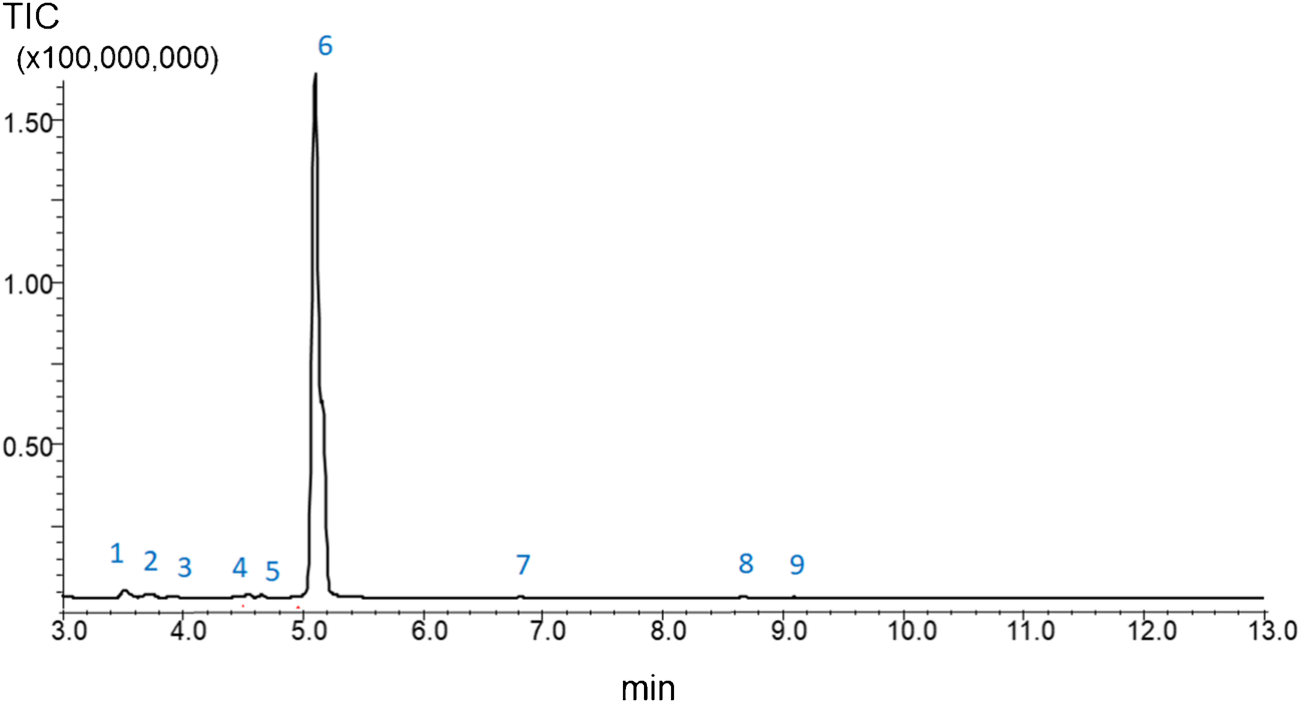
GC–MS chromatogram obtained in the absence of an internal standard for the pristine derivatized supernatant collected after contact with cells for 24 h (OD = 6.1): 1 — 2,3-bis-TMS butane; 2 — TMS ester 2-TMS oxy-propanoic acid; 3 — TMS ester 2-TMS oxy-pentanoic acid; 4 — tris-o-TMS 1,2,3 butane triol; 5 — bis-TMS ether 1-o-heptadecylglycerol; 6 — glycerol-tri-TMS ether; 7 — methyl ester 3,4-bis TMS oxy-benzeneacetic acid; 8 — D-1,2,3,4-tetrakis-O-TMS-ribopyranose; 9 — 1,2,3,4,6-pentakis-O-TMS-alpha-d-glucopyranose. *Note:* EtOH and BuOH possibly present in fermentation samples cannot be visualized in the chromatogram at the used derivatization procedure

The received chromatogram (Fig. 6) and mass spectra of a peak recorded at retention time of 5.12 min (ESI, Fig. S4) clearly indicate the presence of glycerol (after derivatization with MSTFA visualized as glycerol-tri-TMS ether) in the tested biological samples at high amount (Fig. 6). The similarity degree of the defined compounds with a library ranged from 86 to 97%.

#### Impact of interfering species on response of electroplated Pd-modified sensors

**Fig. 7:**
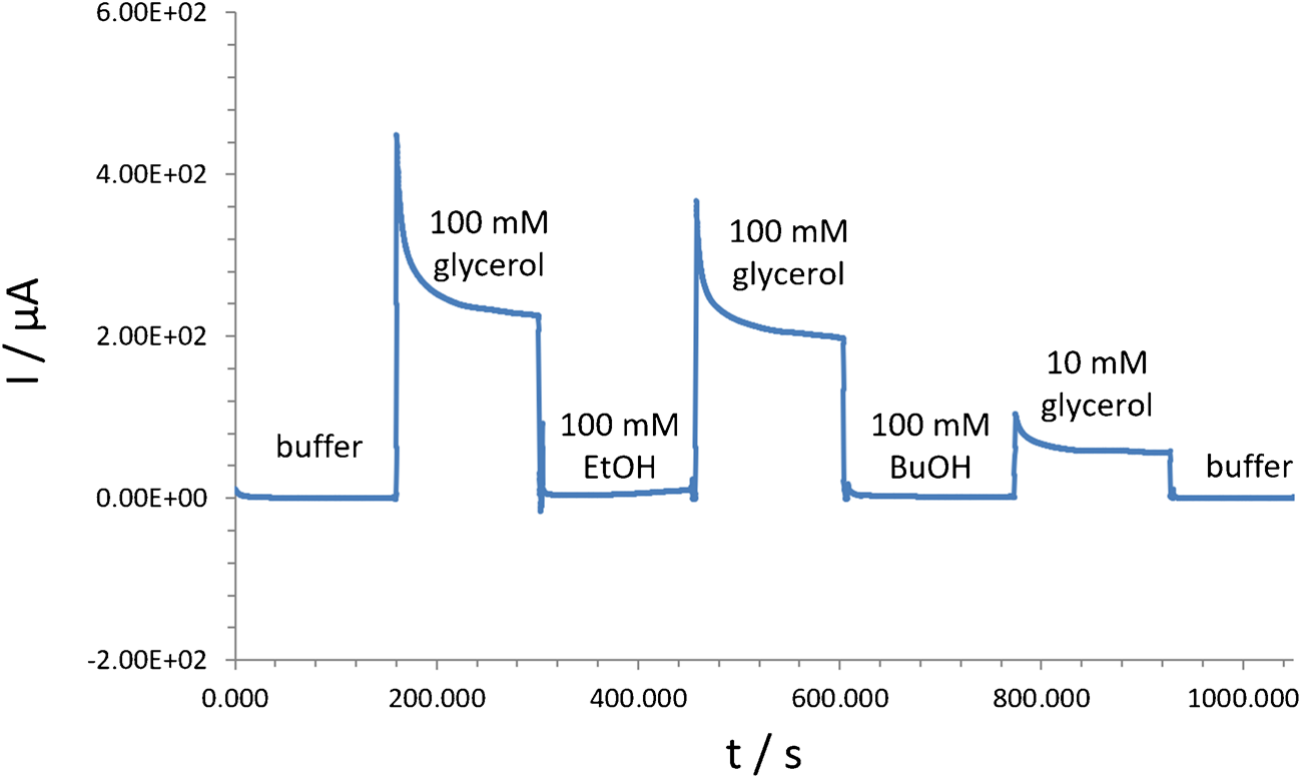
Selectivity test performed with interfering analytes in AM mode from Sensor 2 (as a case study) at the applied potential of 0.55 V vs. Ag/AgCl. *Note:* pH of samples was 12

Further, a selectivity test for the proposed sensors with electroplated Pd layers towards glycerol determination at the defined electrochemical conditions in AM mode was conducted. Notably, sensors with electroplated Pd layers showed the exclusive sensing properties towards glycerol, whereas no response was recorded to interfering species (e.g., EtOH, BuOH) possibly formed during yeast fermentation (Fig. 7). The obtained experimental results can be explained by the advanced adsorption of glycerol at Pd-sensing layers as compared to interfering species, viz. EtOH (see “Impact of the adsorption stage on the glycerol electrooxidation”).

#### Application to glycerol determination in real samples

Finally, the electroanalytical performance of novel sensors with electroplated Pd particles was quantitatively evaluated in a droplet of HC fermentation medium collected after cultivation of yeasts. For this goal, the standard addition approach of glycerol solutions was used. The most sufficient advantage to be mentioned for the used approach is that it does not need a blank matrix for quantification. In addition, this approach overcomes the matrix effects and recovery rates [36, 37].

Concisely, by skipping of fermentation medium taken after 24 h of yeast cultivation, it was revealed that the total glycerol content was in the range of 71–75 mM depending on functional Pd layer used (Table 4). Remarkably, the anodic potential of glycerol electrooxidation was almost identical (i.e., ~ 0.55 to 0.6 V) in a real fermentation sample for all tested sensors (ESI, Fig. S5). Therefore, to simplify data processing, the signal read-out was conducted for all sensors at 0.55 V.

**Table 4:** Electroanalytical results of glycerol quantification in fermentation samples at pH 12 on Pd-modified electrodes polarized from − 0.4 to 0.8 V*

Functional layer	Calibration formula	*R*^2^	Concentration of glycerol, mM	RSD, %	Sensitivity, µA·mM^−1^·cm^−2^
Sensor 1	*y* = 4.16·*x* + 289	0.9949	71 ± 1	1.33	1.54
Sensor 2	*y* = 5.27·*x* + 373	0.9989	72 ± 1	1.90	2.51
Sensor 3	*y* = 7.10·*x* + 537	0.9988	72 ± 1	1.43	2.44
Commercial Pd ink	*y* = 4.59·*x* + 342	0.9799	73 ± 2	2.02	0.49

*Cells with OD = 5.8 obtained after 24 h of cultivation were removed from the medium by centrifugation prior to analysis. The remaining medium was used for the subsequent analysis

Interestingly, the glycerol content in a target fermentation sample was quantified almost at the same level with the same sensitivity regardless of the design of functional Pd layers (Table 4). This observation confirmed the earlier made assumption on the impact of the adsorption stage of glycerol on its electrooxidation at Pd surfaces.

The recovery data obtained in HC medium after yeast cell cultivation for 24 h summarized in Table 5 highlight a strong potential of the proposed assay utilizing sensors with electroplated Pd deposits for glycerol determination even in the presence of interfering species. The concentration of glycerol found by the novel sensors with Pd particles was in line with data received by commercial Pd-ink-modified electrode.

**Table 5:** Selected recovery data for glycerol determination in HC yeast fermentation medium after contact with cells (OD = 5.8)

Electrode/calibration formula	Added glycerol, mM	Found glycerol, mM	Recovery, %	RSD*, %
Sensor 1 *y* = 4.16·*x* + 342	25	25.25 ± 0.65	101.00	2.60
	50	51.31 ± 1.61	102.62	3.11
	75	75.26 ± 2.41	100.34	3.20
Sensor 2 *y* = 5.22·*x* + 373	25	25.33 ± 0.57	101.32	2.11
	50	51.66 ± 2.08	103.32	4.00
	75	75.33 ± 2.30	100.40	3.02
Sensor 3 *y* = 7.10·*x* + 536	25	25.56 ± 0.51	102.24	2.00
	50	49.56 ± 1.52	99.12	3.07
	75	74.78 ± 0.37	99.71	0.49
Commercial Pd-ink *y* = 1.936·*x* + 152	25	24.02 ± 2.74	96.08	11.39
	50	51.98 ± 5.54	103.96	10.67
	75	77.66 ± 2.30	103.54	2.97

*Number of experiments, *n* = 3

Selected quantification data of glycerol present in yeast fermentation samples are summarized in Table S1. Obviously, the glycerol can readily be quantified in yeast supernatants. Next, the optimized assay utilizing electroplated Pd deposits is planned to be used in a tandem work with biochemists and biologists to establish the existing correlations between glycerol content and cultivation conditions of yeast cells.

## Conclusions

Here, by surface engineering investigations with a special focus on the surface design, simple, tuned, reproducible sensing layers consisting of electroplated one-step-produced palladium particles were formed. By optimized electrochemical assay utilizing proposed electroplated Pd-based sensors requiring only a minimum sample preparation (viz. adjusting of pH to 12), glycerol was quantified in yeast fermentation medium with RSD below 3% and recoveries in the range of 99–103%. Received results showed a satisfactory agreement with the control measurement carried out by commercial Pd-ink-based electrode.

More significantly, in terms of fundamental aspects, here for the first time it was demonstrated that the efficiency of glycerol electrooxidation on palladium was not affected by the design of Pd-sensing layers but more likely depended on the adsorption of glycerol.

The obtained knowledge on the impact of surface morphology and engineering of the sensing functional layers on glycerol determination in complex media will assist the developments of the comparative criteria for tracking the design of sensors on their electroanalytical performance in the future.

### Supplementary Information

Below is the link to the electronic supplementary material.

Supplementary file1 (DOCX 368 KB)

## Abbreviations

Pd: Palladium
GC-MS: Gas chromatography-mass spectrometry
SPEs: Screen-printed electrodes
SEM: Scanning electron microscopy
EDX: Energy-dispersive X-ray spectroscopy
MSTFA: N-Trimethylsilyl-N-methyl trifluoroacetamide
OD: Optical density
CV: Cyclic voltammetry
AM: Amperometry
ECSA: Electroactive surface area
DFT: Density functional theory
