# Supplementary material for: Towards controlled and simple design of non-enzymatic amperometric sensor for glycerol determination in yeast fermentation medium
Source: Anal Bioanal Chem. 2024 May 3;416(15):3619–30. doi: 10.1007/s00216-024-05316-7 (PMC11156751; doi:10.1007/s00216-024-05316-7)
Supplement: Supplementary file 1 — Supplementary file1 (DOCX 368 KB) [file 216_2024_5316_MOESM1_ESM.docx]

**Supplementary Materials**

**Towards controlled and simple design of non-enzymatic amperometric sensor for glycerol determination in yeast fermentation medium**

E.V. Zolotukhina^1^, E.V. Butyrskaya^2^, C. Fink-Straube^3^, M. Koch^3^, Y.E. Silina^4*^

*^1^Federal Research Center of Problems of Chemical Physics and Medicinal Chemistry,*

*Russian Academy of Sciences, Сhernogolovka,* *Moscow region, Russia*

*^2^Department of Chemistry, Voronezh State University, Voronezh, Russia*

*^3^INM – Leibniz Institute for New Materials, Saarbrücken, Germany*

*^4^Institute of Biochemistry, Saarland University, Saarbrücken, Germany*

*^*^The correspondence*

*should be addressed (Y.E. Silina)*

*Institute of Biochemistry,*

*Saarland University, Saarbrücken, Germany*

*Campus B 2.2, room 317*

*E-mail 1: yuliya.silina@gmx.de*

*E-mail 2:* [*yuliya.silina@uni-saarland.de*](mailto:yuliya.silina@uni-saarland.de)

**
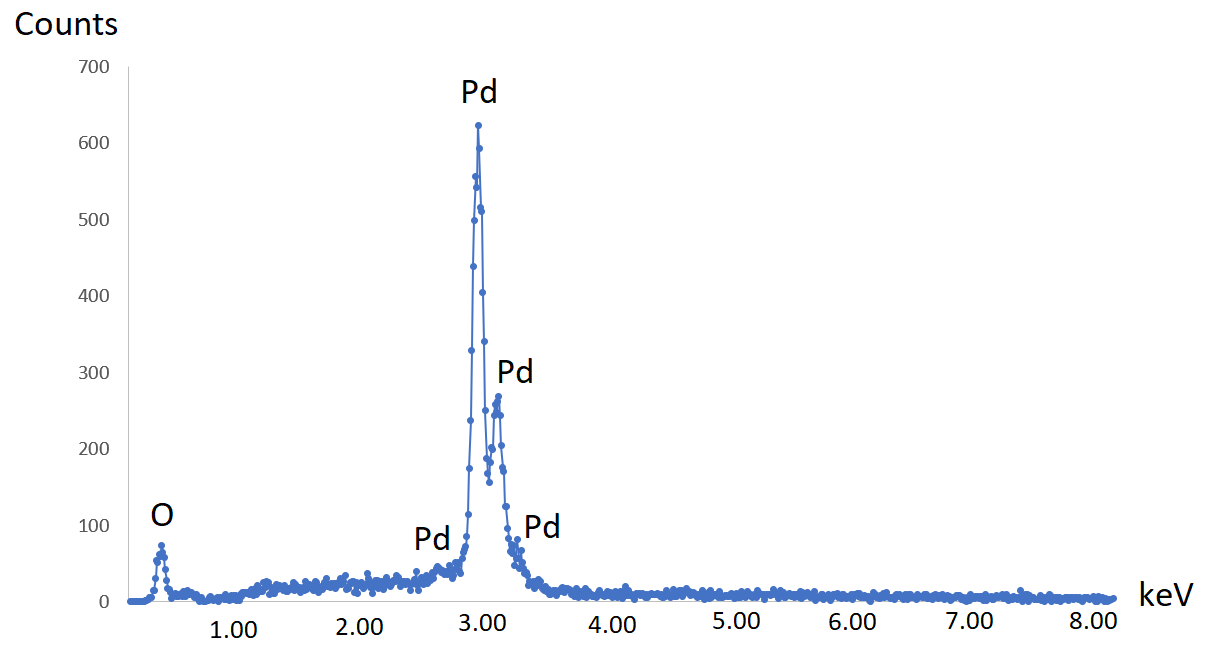
**

**Fig. S1** – EDX spectra received from *Sensor 3* (Pd-particles deposition at -6 mA for 240 s).

**Fig. S2** – Overlaid CV plots recorded from Pd-based sensors at 20 mV/s in 100 mM of model glycerol solution at pH 12: *1* – commercial Pd-ink modified electrode; *2 – Sensor 1*; *3 – Sensor 2*; *4 – Sensor 3.*

**
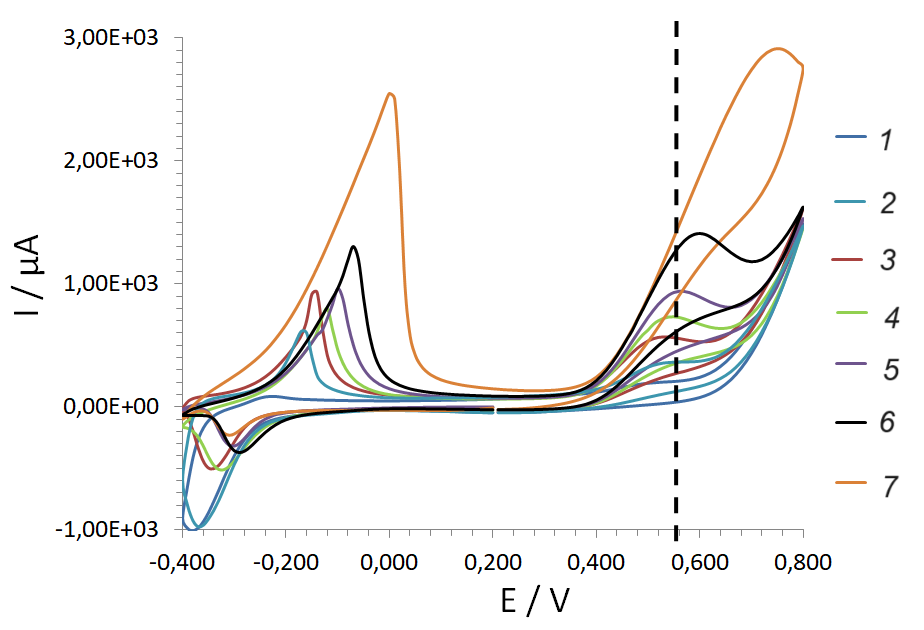
**


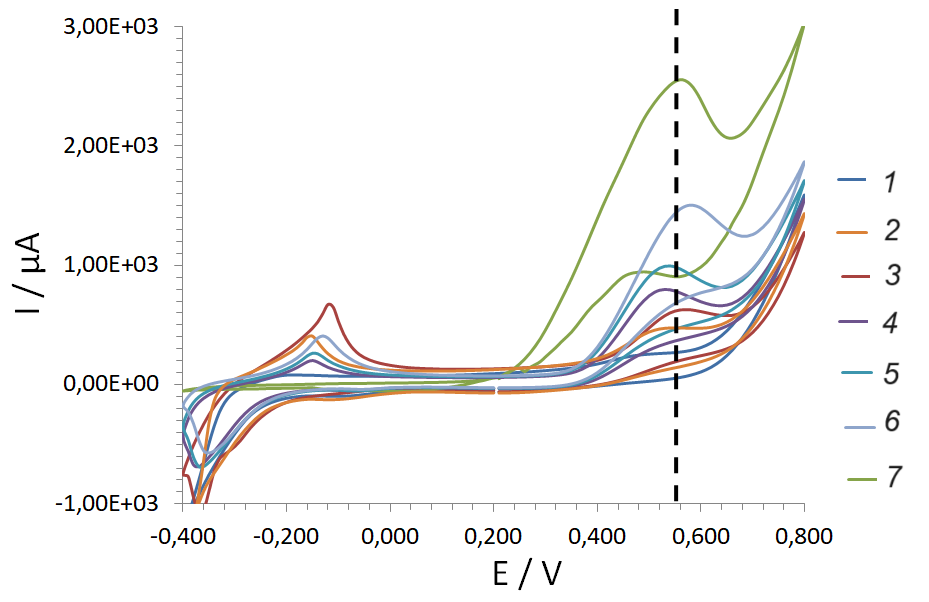


**Fig. S3** – CV plots recorded in model glycerol solution at pH 12 from *Sensor 1 (top)* and *Sensor 2 (bottom)* at 20 mV/s: *1*,*2*,3*,4,5,6,7* – 1 mM, 5 mM,10 mM, 15 mM, 20 mM, 50 mM, 100 mM, respectively.


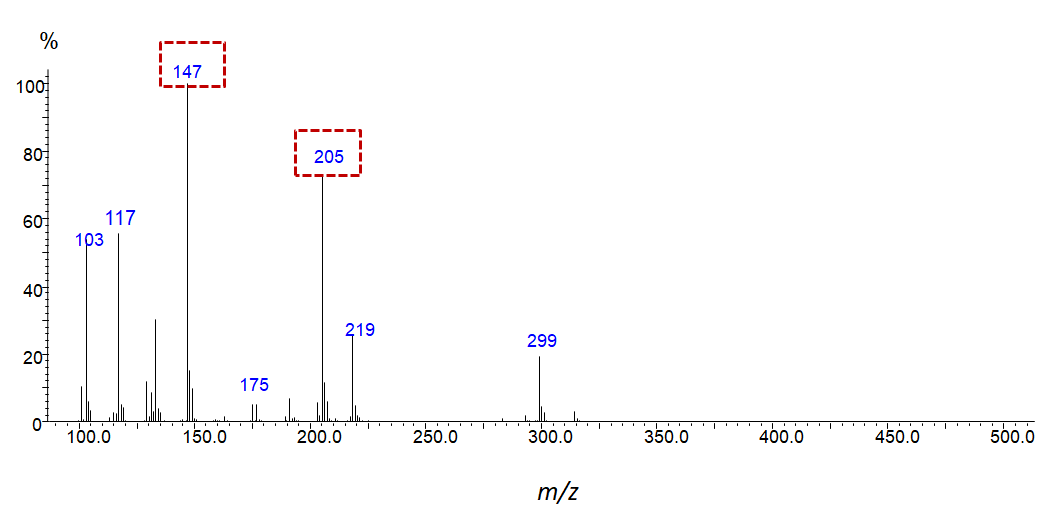


**Fig. S4** – GC-MS mass spectra recorded at RT = 5.12 min and corresponding to glycerol-tri-TMS ether. *Note:* type and distribution of fragments at *m/z* 205 and *m/z* 147 is typical for glycerol, 3TMS derivative.

|  |  |
| --- | --- |
|  |  |

**Fig. S5** – CV plots recorded in fermentation sample (OD = 5.8) at 20 mV/s and pH 12 from the surface of: **A** – *Sensor 1*, **B** – *Sensor 2*, **C** – *Sensor 3*; **D** – Pd-ink modified electrode.

**Table S1** – Content of glycerol in yeast fermentation samples/supernatants determined by novel sensors with electroplated Pd-layers (shown for *Sensor 2* as a case study)

| Sample | Calibration formula | R^2^ | Found glycerol concentration, mM |
| --- | --- | --- | --- |
| Fermentation medium after contact with cells (OD = 6.1) | y = 1.496·x + 106.6 | 0.9956 | 74 ± 0.65 |
| Fermentation medium after contact with cells (OD = 6.8) | y = 2.584·x + 239.0 | 0.9989 | 78 ± 1.12 |
